# The Vasoactive Potential of Kisspeptin-10 in the Peripheral Vasculature

**DOI:** 10.1371/journal.pone.0014671

**Published:** 2011-02-09

**Authors:** Iain Sawyer, Sarah-Jane Smillie, Jennifer V. Bodkin, Elizabeth Fernandes, Kevin T. O'Byrne, Susan D. Brain

**Affiliations:** 1 Vascular Biology Section, Cardiovascular Division, King's College London, British Heart Foundation Centre, London, United Kingdom; 2 Division of Women's Health, King's College London, London, United Kingdom; Istituto Dermopatico dell'Immacolata, Italy

## Abstract

Splice products of the Kiss1 protein (kisspeptins) have been shown to be involved in a diverse range of functions, including puberty, metastasis and vasoconstriction in large human arteries. Circulating Kisspeptin-10 (Kp-10) plasma levels are low in normal individuals but are elevated during various disease states as well as pregnancy. Here, we investigated the potential of Kp-10, the shortest biologically active kisspeptin, to influence microvascular effects, concentrating on the cutaneous vasculature. Kp-10 caused a dose-dependent increase in oedema formation (0.3–10nmol/injection site), assessed by Evans Blue albumin dye extravasation, in the dorsal skin of CD1 mice. Oedema formation was shown to be inhibited by the histamine H_1_ receptor antagonist mepyramine. The response was characterised by a ring of pallor at the injection site in keeping with vasoconstrictor activity. Therefore, changes in dorsal skin blood flow were assessed by clearance of intradermally injected ^99m^technetium. Kp-10 was found to significantly reduce clearance, in keeping with decreased blood flow and providing further evidence for vasoconstrictor activity. The decreased clearance was partially inhibited by co-treatment with the cyclo-oxygenase inhibitor indomethacin. Finally evidence for the kisspeptin receptor gene (*Kiss1R*), but not the kisspeptin peptide gene (*Kiss1*), mRNA expression was observed in heart, aorta and kidney samples from normal and angiotensin II induced hypertensive mice, with similar mRNA levels observed in each. We have evidence for two peripheral vasoactive roles for kisspeptin-10. Firstly, plasma extravasation indicative of ability to induce oedema formation and secondly decreased peripheral blood flow, indicating microvascular constriction. Thus Kp-10 has vasoactive properties in the peripheral microvasculature.

## Introduction

The *Kiss1* gene encodes a 145-amino acid protein that is cleaved into a 54-amino acid peptide known as kisspeptin-54 (Kp-54) as well as shorted peptides of 14, 13 or 10 amino acids [1]. These peptides, collectively referred to as kisspeptin, have a wide range of functions in different tissues and physiological processes. Upon its discovery in 1996, Kp-54, also known as metastin, was found to inhibit metastasis of malignant melanoma cells [2] which has since been replicated in breast cancer cells treated with Kp-10(Kp-10) [3]. This ability to inhibit the migration of cells was further confirmed when the movement of primary trophoblasts, crucial for placental development during pregnancy, was halted by Kp-10 treatment [4]. However, the most important role of kisspeptin is currently considered to be its control of reproductive function. Inactivating mutations of the Kp-10 receptor (Kiss1R, also known as GPR54) in humans are associated with failure to progress through puberty and adult infertility (hypogonadotropic hypogonadism) [5], [6]. Similar defects were found in GPR54 knockout mice [5]. Kp-10 stimulates secretion of the gonadotropic hormones luteinising hormone (LH) and follicle-stimulating hormone (FSH) [7] via a direct action on the hypothalamic gonadotropin releasing hormone (GnRH) neurones which contain Kiss1R [8]. Not only is Kp-10 the most potent activator of gonadotropic hormone secretion, but it plays a key role in regulating GnRH pulse generator frequency a critical component controlling the hypothalamo-pituitary-gonadal axis [9].

Recently it has been suggested that members of the kisspeptin family may also possess vasoactive activity. Kp-10 has been shown to inhibit the migration of human umbilical vein endothelial cells and subsequent angiogenesis through inhibition of vascular endothelial growth factor (VEGF) signalling [10]. Additionally, Mead *et al* (2007) identified transcription of both *Kiss1* and *Kiss1R* mRNA in human aorta, umbilical vein and coronary artery but not other vessels [11]. It was further suggested that Kp-10, -13 and -54 may be novel vasoconstrictors *in vivo* as all three peptides resulted in contraction of human vessels in myograph studies with comparable potency to angiotensin II (Ang II), a potent vasoactive peptide [11]. Also, endothelial cells were postulated to be an alternative source of kisspeptin, possibly contributing to circulating plasma levels or acting in a local paracrine fashion. To date, studies have been confined to large conductance vessels. By comparison, the smaller vessels of the microvasculature are important for controlling tone and other vasoactive effects, where increases in permeability to plasma proteins lead to plasma extravasation and oedema formation.

Circulating plasma Kp-10 levels in normal individuals are believed to be low. However, the concentration of Kp-10 increases from 1.3fmol/ml pre-pregnancy to 9.6pmol/ml in the third trimester [12]. The source of this increase is believed to be the placenta, as Kp-10 levels drop almost immediately after birth [12]. The vascular effects of Kp-10 may be important as hypertension and oedema are symptoms of pre-eclampsia prevalent in late-term pregnancies but not after birth [13]. It has been shown that trophoblasts from women with pre-eclampsia have significantly higher kiss1 mRNA and Kp-54 peptide levels than trophoblasts during normal pregnancies [14]. By comparison, contrasting data has also been published suggesting that serum from women with pre-eclampsia contain less Kp-10 than control individuals [15]. At present it is known that circulating levels are sufficient to influence vascular function in physiological situations. However, kisspeptin's effect in either situation where circulating levels are high or where there are high local levels, as observed within the placenta during late pregnancy, may well influence local vasoactive events. With this concept in mind we have utilised established procedures for assessing microvascular blood flow and plasma extravasation in the cutaneous microvasculature of laboratory mice to investigate the ability of Kp-10 to influence vasoactive effects at the microvascular level. We have previously shown in studies of the cutaneous microvasculature and in similar *in vivo* assays that i) calcitonin gene-related peptide (CGRP) is one of the most potent vasodilators known [16] ii) endothelin-1 (ET-1) has the ability to mediate microvascular vasoconstriction [17] and iii) alarin (a novel member of the galanin family of peptides) mediates vasoconstrictor activity [18].

Here, we investigated the microvascular effects of Kp-10, the shortest biologically active proteolysis product of the *Kiss1* gene, in the peripheral microvasculature of mice. The study was controlled through comparison with the known microvascular activities of the neuropeptides substance P, CGRP and endothelin-1.

## Materials and Methods

### Ethics

Experiments were carried out under the U.K. Animals (Scientific Procedures) Act, 1986 under licence # PPL 70/6899. Procedures were also approved by the Kings College London local ethical review committee. All animals were maintained on a normal diet, with free access to food and water, in a climatically controlled environment.

### Animals

Female and male CD1 and C57BL/6 mice (approximately 25g, 8–12 weeks old) were obtained from Charles River (Kent, U.K.).

### Reagents

All reagents were purchased from Sigma-Aldrich (U.K.) unless specified. Human Kp-10 was purchased from Alta Bioscience (Birmingham, U.K.). CGRP was purchased from Cambridge Bioscience (Cambridge, U.K.). All peptides were dissolved in distilled water and stored at −20°C or −80°C until further dilution in saline immediately prior to use.

### Measurement of Plasma Extravasation

Mice were anaesthetised with urethane (25% w/vol; 2.5 g/kg i.p.), and the dorsal skin was shaved. Plasma extravasation was used as an indication of oedema formation and assessed using an Evans Blue accumulation assay in skin, as described by Cao *et al* (1999) [19]. Mice received Evans Blue (25% w/vol; 2.5mg/g i.v.) and after 3 minutes plasma extravasation was induced by an intradermal injection (50µL per site) containing saline, Kp-10 (0.3–10nmol) substance P (300pmol), CGRP (10pmol), histamine (20nmol), p234 (1nmol) or mepyramine (5nmol). In order to prevent site-related effects, injection sites were randomised on the dorsal skin. Plasma extravasation was quantified at different time points (7.5, 15 or 30 minutes) through pre-injecting at these time points prior to cervical dislocation. The diameter of the blue lesion was measured twice, at 90°C angles in order to provide an approximate area as previously described [20]. Each site was also assessed for intensity of blueness using a qualitative score of 0 (no blue, thus no evidence of plasma extravasation) to 5 (darkest blue indicative of highest plasma extravasation). The mean of the results for each site was determined and results for the quantitative diameter assay to show spread of plasma extravasation and the qualitative assay to show the intensity of the plasma extravasation are shown.

### Assessment of Changes in Blood Flow

Blood flow was assessed using a clearance technique in skin, as Schmidhuber *et al* (2007) [21]. Test agents containing an equal amount of radioactive ^99m^technetium (^99m^Tc) were prepared immediately prior to use. Saline, Kp-10 (3–10nmol), endothelin-1 (30pmol) or indomethacin (3nmol) were co-injected with an equal amount of radioactive ^99m^technetium (^99m^Tc; approx. 20kBq/site i.d.; Nuclear Medicine , Guy's Hospital, London) in the dorsal skin of male and female CD1 mice (50µL per site). An equal volume of agent(s) under test + ^99m^Tc was used for measurement of total radioactivity. A 10 min clearance period was used, where radioactivity cleared from the skin in proportion to blood flow through the skin [21]. The clearance period was instantly terminated by cervical dislocation and death, which reduced blood flow to zero. The injected sites were then rapidly punched out (11mm diameter) and the remaining radioactivity immediately counted in an adjacent radioactive gamma counter. Clearance was calculated by comparison of paired skin punch and total radioactivity readings. Test agent specific clearance was then calculated by comparison to saline controls, normalised to 100%, and expressed as % change in clearance compared to control. Positive values are due to decreased clearance that is directly related to decreased blood flow and vasoconstrictor activity.

### Mouse Model of Angiotensin II Induced Hypertension

In order to possibly learn more of the potential of Kp-10 to influence cardiovascular dysfunction, gene expression was evaluated in different tissues collected from hypertensive animals. Hypertension was induced as described by Liang *et al* (2009) [22]. Briefly, Alzet osmotic minipumps (Charles River, U.K.) with a 14 day capacity, average volume of 100µL and an infusion rate of 0.25µl/hour were filled with saline or angiotensin II (adjusted to mouse weight to deliver 1.1mg/kg/day, a dose previously shown to induce hypertension [23] were implanted subcutaneously in wild type (WT) female C57BL/6 mice in the mid-scapular region under isoflurane anaesthesia. Mice were terminated after 14 days by cervical dislocation and heart, aorta and kidneys collected. Vehicle treated animals were used as controls. Tissues were immediately immersed in RNAlater (Ambion) for 24 hours, snap-frozen and stored at −80°C until analysis.

### Quantitative mRNA Expression Analysis

Expression of *Kiss1* and *Kiss1R* mRNA in homogenised tissues was determined by real-time quantitative PCR using the Syber Green method. Briefly, total RNA was extracted from the tissues using the RNeasy Fibrous Tissue kit and (Qiagen, U.K.) and reverse transcribed to cDNA using the High Capacity RNA-to-cDNA kit (Applied Biosystems) according to the manufacturer's instructions. Real-time PCR was performed using the Sensimix SYBER One Step Kit (Bioline). The following primers were chosen and designed previously (Sigma) [24]: mouse Kiss1 sense: ttcttggcagctgctgctt, antisense: cgaaggagttccagttgtag (product: 319 bp, T_ann_ 55°C); mouse Kiss1R: sense: ggctccgtccaacgcttcag, antisense: tgtgcttgtggcggcagata (product 175 bp, T_ann_ 58°C). Results are expressed as copy number and normalised by comparison to reference genes HPRT-1, SDHA and PLA2 using GeNorm version 3.4. All experiments were performed in accordance with the MIQE guidelines [25].

### Statistical Analysis

Results are displayed as mean ± SEM. Statistical analysis was performed using one way ANOVA followed by a Dunnet's or Bonferroni's multiple comparison test or a paired two-tailed Student's *t*-test as indicated. Significance was accepted when p≤0.05. *N* represents the number of animals used in each experiment.

## Results

### Kp-10 Caused a Dose-Dependent Increase in Plasma Extravasation

**Figure 1:**
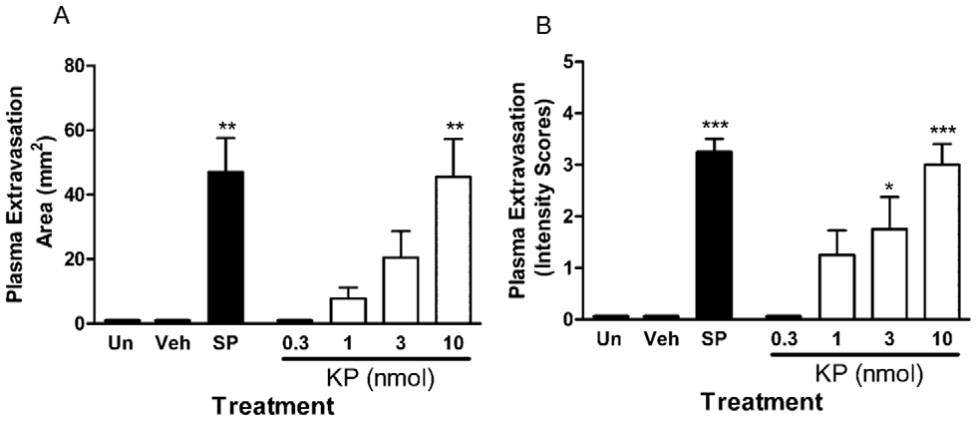
Kisspeptin-10 causes a dose-dependent increase in oedema formation in the cutaneous vasculature. Female CD1 mice were anaesthetised, injected with Evans Blue dye i.v. and treated intradermally in the dorsal skin with 0.3–10nmol kisspeptin-10 (KP), 300pmol substance P (SP) or saline control (Veh) for 30 minutes. An uninjected site was also included (Un). Plasma exudation was allowed to continue for 30 minutes post-injection at which point mice were killed, dorsal skin removed and the area of oedema measured (A). The lesion was also scored for colour intensity (B). Results are shown as mean area (mm^2^) ± SEM, *N* = 4. * = p≤0.05, ** = P≤0.01, *** = p≤0.001 assessed by one-way ANOVA compared to vehicle control.

**Figure 2:**
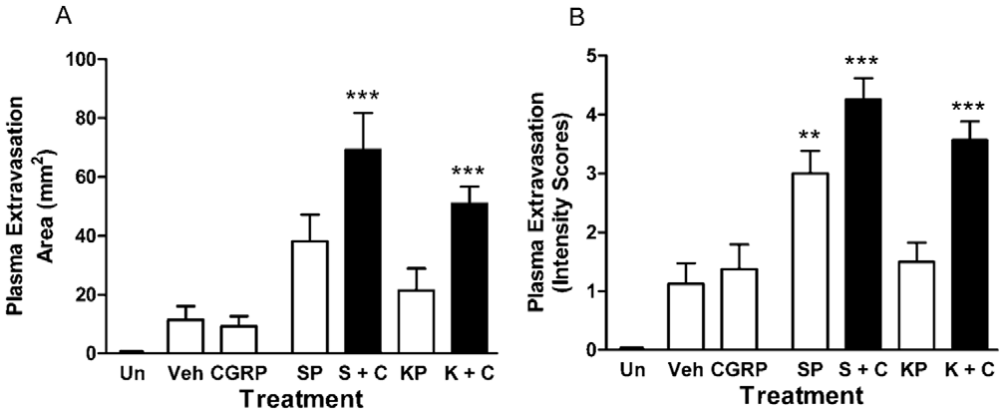
Kp-10 induced oedema is potentiated by CGRP. Female CD1 mice were anaesthetised, injected with Evans Blue dye i.v. and treated intradermally in the dorsal skin with 10pmol CGRP, 300pmol substance P (SP), 3nmol Kp-10 (KP), 10pmol CGRP +3pmol SP (S + C), 3nmol Kp-10 +10pmol CGRP (K + C) or saline control (Veh) for 30 minutes. An uninjected site was also included (Un). Plasma exudation was allowed to continue for 30 minutes post-injection, mice were killed, dorsal skin removed and the area of oedema measured (A). Lesion colour intensity was also assessed (B). Results are shown as mean area (mm^2^) ± SEM. *N* = 8. * = p≤0.05, ** = p≤0.01, *** = p≤0.001 assessed by one-way ANOVA compared to vehicle control.

Increases in plasma extravasation and subsequent oedema were assessed by an Evan's Blue dye technique. This dye binds to albumin present in plasma following i.v. injection and exudation after intradermal injection of vascular permeability increasing agents is observed as a blue ring in the skin. The measurement of this can be either as diameter of blueing, to show the spread of plasma extravasation, or as intensity to give an estimation of the amount of leakage. As expected, substance P (300pmol/site) but not CGRP (10pmol/site) caused significant plasma extravasation in the dorsal skin (Figures 1 & 2) [19]. Substance P-induced plasma extravasation was potentiated by co-treatment with the vasodilator CGRP (Figure 2). A dose-dependent increase in plasma leakage was observed in Kp-10 treated animals (0.3–10nmol/site) as assessed by area after 30 minutes (Figure 1). Plasma extravasation was observed at 1–3nmol/site Kp-10 but was not significant. However, treatment with 10nmol/site Kp-10 produced significant plasma extravasation similar to that of the well characterised permeability agent substance P. Additionally, the plasma extravasation caused by Kp-10 (3nmol/site; 30 min) was potentiated by the co-injection with CGRP (10 pmol/site) (Figure 2). The similarity between the two different types of measurements is noted.

### Kisspeptin-Mediated Plasma Extravasation is an Acute Response

To assess the duration of action of kisspeptin-mediated plasma extravasation formation, mice were treated i.d. with test agents and the experiment terminated after 7.5, 15 or 30 minutes. Plasma extravasation was then assessed as previously described (Table 1). As expected, Kp-10 (0.3–10nmol/site) induced a dose-dependent plasma extravasation at all evaluated time points. The onset of plasma extravasation was visible in less than 7.5 minutes. Minor increases in permeability were observed after 15 minutes but clearance of 1 and 3 nmol/site Kp-10 mediated plasma extravasation may have occurred between 15 and 30 minutes post-treatment leading to decreases in plasma extravasation area. No difference in plasma extravasation was observed between time points when Kp-10 was injected at 10 nmol/site, which produced the highest observed levels of plasma extravasation.

**Table 1:** Kisspeptin-10 mediated oedema formation is visible 7.5 minutes post-injection.

	Time (Minutes)		
KP (nmol/site)	7.5	15	30
0.3	5.20±1.74	23.5±8.84	0±0
1	20.8±4.75**	21.7±5.30	7.75±3.50
3	39.2±4.26***	43.7±10.7***	20.5±8.13
10	38.4±10.5***	47.8±6.41***	45.5±11.7*

Female CD1 mice were anaesthetised, injected with Evans Blue i.v. and treated intradermally with 0.3–10nmol Kp-10 (KP) in the dorsal skin for 7.5, 15 or 30 minutes. Mice were killed, the dorsal skin removed and the area of oedema measured. Results are shown as mean area (mm^2^) ± SEM (3 s.f.). 7.5, 15 and 30 minute treatment groups consisted of *N* = 5, *N* = 6 and *N* = 4 respectively.

* = p≤0.05,

** = p≤0.01,

*** = p≤0.001 compared to vehicle controls as assessed by one way ANOVA followed by a Dunnet's post test.

### Inhibitor Effect of a Histamine Antagonist on Kp-10 Induced Plasma Extravasation

**Figure 3:**
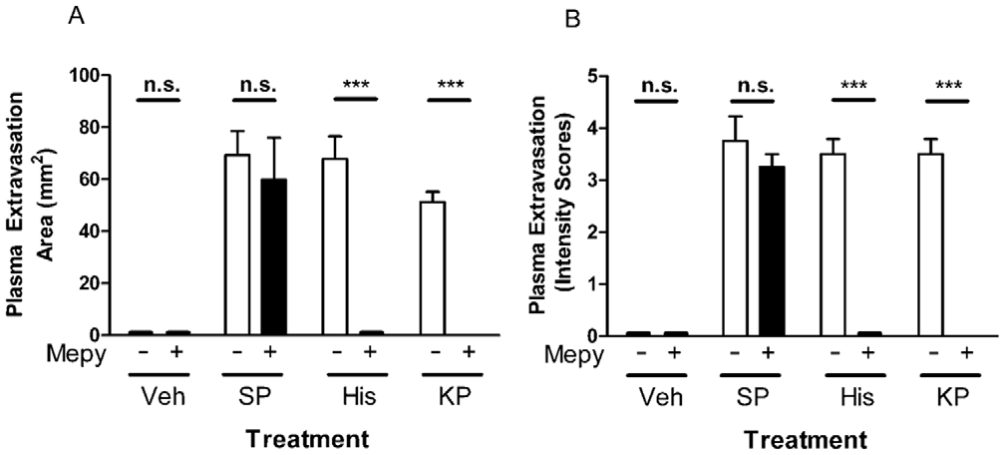
Mepyramine inhibits Kp-10 induced oedema formation. Female CD1 mice were anaesthetised, injected with Evans Blue i.v., and then exposed intradermally to saline (Veh), 20nmol Histamine (His), 300pmol substance P (SP) and 10nmol Kp-10 (KP) +/−5nmol Mepyramine (Mepy) for 15 minutes. Mice were then killed, dorsal skin removed and the oedema area measured (A). Lesion colour intensity was also assessed (B). Co-treatment with saline is indicated by white bars and co-treatment with mepyramine is indicated by black bars. Results shown as mean area (mm^2^) ± SEM, *N* = 4. n.s. = no significant difference between treatments, *** = p≤0.001 between mepyramine untreated and treated groups as assessed by two way ANOVA.

In order to investigate the role of mast cell released histamine in Kp-10 mediated plasma extravasation formation, female CD1 mice were co-treated intradermally with Kp-10 (10nmol/site), histamine (20nmol/site) or substance P (300pmol/site) and mepyramine (5nmol/site), a histamine H_1_ receptor antagonist [26]. Histamine, substance P and Kp-10 injection caused significant plasma extravasation formation in the dorsal skin 15 minutes post-injection when compared to control (saline) (Figure 3). Substance P-treated sites were unaffected by co-treatment with mepyramine. However, in the presence of mepyramine both histamine- and Kp-10- mediated plasma extravasation was abolished.

An attempt was made to inhibit Kp-10-induced plasma extravasation using p234, a Kp-10 derived antagonist of Kiss1R [27]. However, when injected at 1 nmol/site, p234 caused a marked plasma extravasation with an area of 60.7±5.3mm^2^ (*N* = 3). This plasma extravasation was also abolished by co-treatment with 5nmol/site mepyramine.

### Kp-10 Induced Plasma Extravasation Presents with a Pallor Ring at the Injection Site

**Figure 4:**
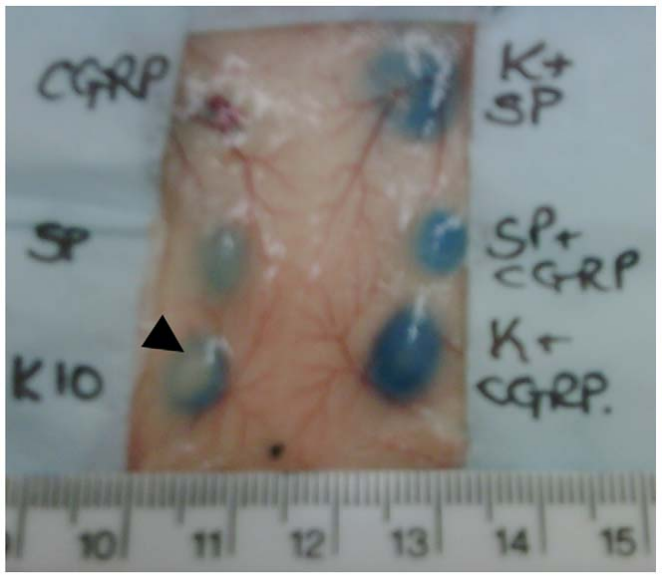
Kisspeptin induced oedema presents with a pallor ring at injection site in the dorsal skin. Female CD1 mice were anaesthetised, injected with Evans Blue i.v. and treated intradermally in the dorsal skin with 10pmol CGRP, 300pmol substance P (SP), 10nmol Kp-10 (K10), 10nmol Kp-10 +300pmol substance P (K + SP), 300pmol substance P +10pmol CGRP (SP + CGRP) or 10nmol Kp-10 +10pmol CGRP (K + CGRP). Plasma extravasation was allowed to continue for 30 minutes. Mice were killed, the dorsal skin removed and photographed. Pallor ring present in Kp-10 mediated oedema, but not under other conditions, is indicated.

**Figure 5:**
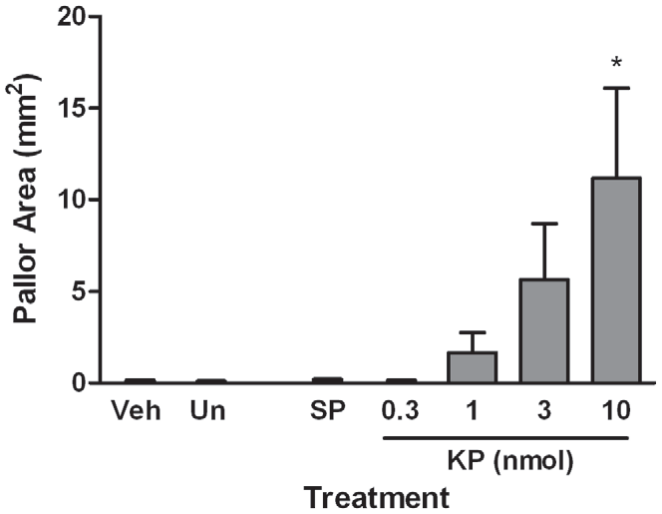
Pallor inhibition ring at centre of kisspeptin mediated oedema increases in a dose dependent manner. Female CD1 mice were anaesthetised, injected with Evans Blue i.v. and treated intradermally in the dorsal skin with 0.3–10nmol Kp-10 (KP), 300pmol substance P (SP) or saline control (vehicle) in the dorsal skin. An uninjected site was also included (Un). Plasma extravasation was allowed to continue for 15 minutes. The skin was removed and the region in which oedema was inhibited (pallor area) measured. *N* = 6. Results are shown as mean area (mm^2^) ± SEM. * = p≤0.05, as assessed by one-way ANOVA compared to vehicle control.

Upon observation of the oedema mediated by Kp-10 treatment it was clear that, unlike substance P induced plasma extravasation, a pallor ring was present at the injection site (Figure 4). This region of plasma extravasation inhibition was measured 7.5 and 15 minutes post-treatment (Figure 5). After 7.5 minutes this area of plasma extravasation inhibition was visible in both 3 and 10nmol Kp-10 treated injection sites, where they were of equal magnitude. However, after 15 minutes of plasma leakage the pallor size of the 10nmol Kp-10 treatment had increased, whereas the inhibition observed at 3nmol had not. A small area of inhibition was present in 1nmol Kp-10 injection sites after 15 minutes but this was not significant.

### Kp-10 Decreased Blood Flow in the Peripheral Vasculature

**Figure 6:**
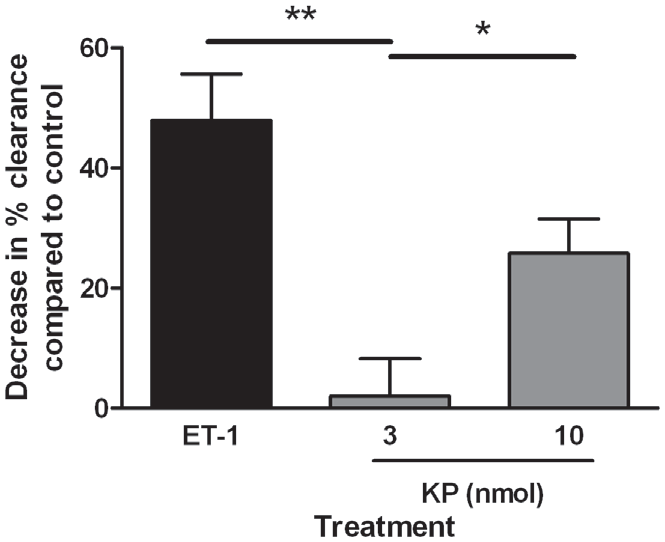
Kisspeptin-10 decreases blood flow in the peripheral vasculature. Male and female CD1 mice were anaesthetised, injected intradermally via the dorsal skin with 3 or 10nmol KP-10 (KP) or 30pmol Endothelin-1 (ET-1), each containing an equal amount of ^99m^Tc. Clearance after ten minutes was assessed and compared to total radioactivity in an uninjected sample. Saline vehicle was adjusted to 100% and results displayed as a % change to controls. Results are shown as mean ± SEM, *N* = 5. * = p≤0.05, ** = p≤0.01 as assessed by two-tailed Student's t-test.

Mice were injected intradermally with Kp-10 (3–10 nmol/site), endothelin-1 (30 pmol/site) or vehicle containing equal amounts of ^99m^Tc. After ten minutes, animals were culled and the skin samples were obtained. The remaining radioactivity in each injection site was compared to a control sample of total radioactivity injected into a tube (Figure 6). Endothelin , an established vasoconstrictor agent [28], endothelin-1 caused a significant decrease in clearance from the injection site (60% decrease from vehicle at a dose of 30pmol, p≤0.01. At 3 nmol/site, Kp-10 treatment resulted in a negligible decrease in clearance compared to vehicle control. However, at 10 nmol/site Kp-10 caused a significant decrease in blood flow from 3 nmol/site suggesting vasoconstrictor activity in the peripheral vasculature (p≤0.05).

### 3.6. Indomethacin Reduced Kp-10 Induced Decreased Blood Flow

**Figure 7:**
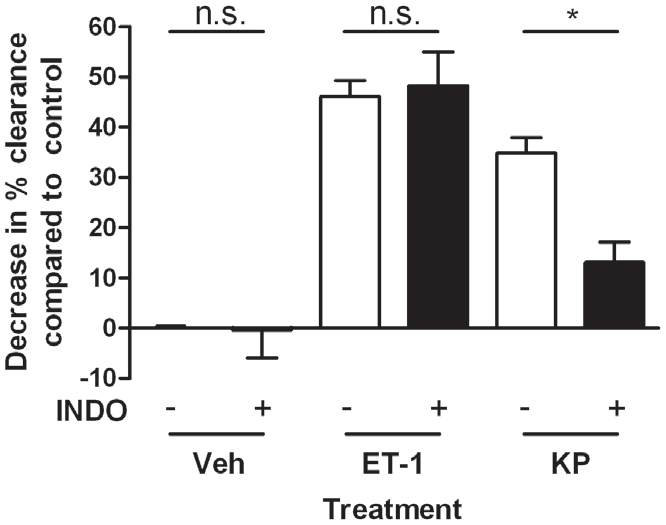
Indomethacin inhibits kisspeptin-induced vasoconstriction. Female CD1 mice were anaesthetised, injected intradermally via the dorsal skin with 5% NaHCO_3_/saline (Veh), 10nmol KP-10 (KP) or 30pmol Endothelin-1 (ET-1) in the presence or absence of 3nmol indomethacin (INDO), each containing an equal amount of ^99m^Tc. Clearance after ten minutes was assessed and compared to total radioactivity in an uninjected sample. Saline vehicle was adjusted to 100% and results displayed as a % change to controls. Results are shown as mean ± SEM, *N* = 6. * = p≤0.05 as assessed by one way ANOVA followed by a Bonferroni's post test.

To investigate the mechanisms underlying Kp-10induced decreases in peripheral blood flow, female CD1 mice were co-treated intradermally with vehicle (5% NaHCO_3_/saline), 30pmol/site endothelin-1 or 10nmol/site Kp-10 in the presence or absence of 3nmol/site indomethacin, an inhibitor of cyclooxygenase, containing equal amounts of ^99m^Tc (Figure 7). After ten minutes, indomethacin alone caused no change in blood flow compared to vehicle. As previously demonstrated, 30pmol/site endothelin-1 caused a significant decrease in blood flow which was not shown to be altered by co-treatment with indomethacin. However, 10nmol/site Kp-10 induced vasoconstriction was significantly reduced (p≤0.05), but not totally inhibited, by co-treatment with indomethacin.

### Kiss1R mRNA, but not Kisspeptin, is Expressed in the Kidney, Heart and Aorta

**Figure 8:**
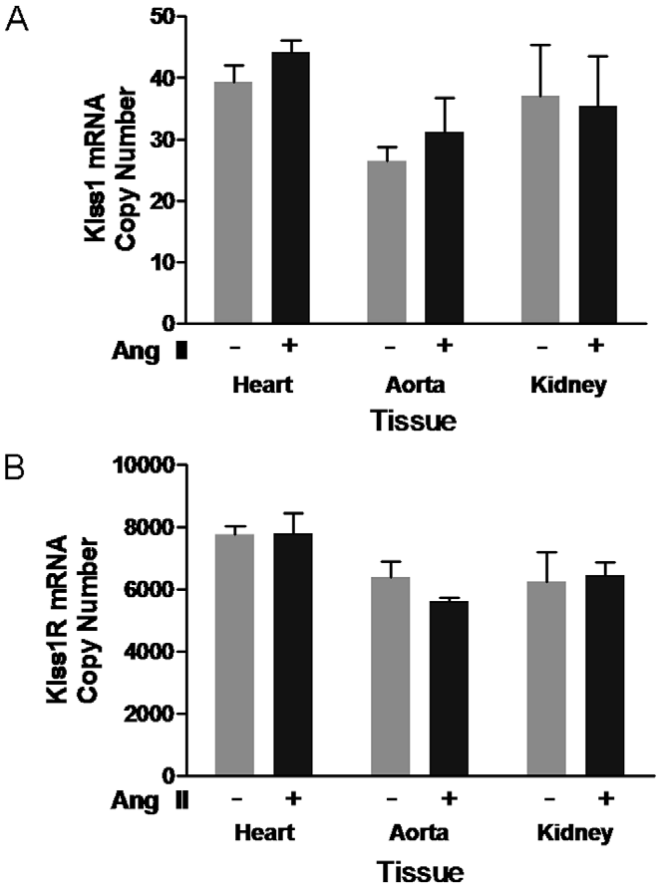
Kisspeptin and Kiss1R expression does not alter after Angiotensin II treatment. C57BL/6 mice were treated with saline (white bars) or angiotensin II (Ang II, black bars) for 14 days. They were then killed and their hearts, kidneys and aortas removed before *Kiss1* (A) and *Kiss1R* (B) mRNA expression analysis. Results are displayed as mean copy number ± SEM normalised against HPRT-1, SDHA and PLA2 reference genes. *N* = 3 per group. No significant difference was observed between groups.

C57BL/6 mice were infused with 1.1mg/kg/day angiotensin II, a dose previously shown to significantly increase systolic blood pressure and heart-to-body weight ratio [23]. In this case, hypertensive animals exhibited systolic pressure readings of 129±3.8mmHg compared to controls (118±0.99mmHg). Hearts, aortas and kidneys were removed and analysed for *Kiss1* and *Kiss1R* mRNA expression (Figure 8). Kp-10 mRNA was expressed at low copy numbers under both conditions, unlike Kiss1R which was expressed at higher levels in all three tissues but did not increase after exposure to angiotensin II.

## Discussion

Kisspeptins have been shown to possess well characterised responses in puberty and reproduction [29], but little is known about their cardiovascular effects. It has been suggested that kisspeptins may be potent vasoconstrictor compounds [11]. This study presents evidence that kisspeptins have the potential to mediate effects in the peripheral microvasculature.

A novel insight into Kp-10 biology gained during this study was the ability of Kp-10 to increase plasma extravasation, measured by both the spread of the plasma extravasation, and through an indication of intensity of staining, determined following Evans Blue accumulation. This was an acute response, visible at 7.5 minutes post-treatment, increasing in a dose-dependent manner, but requiring approximately a 30 times greater dose than that for substance P to elicit a similar plasma extravasation area. The plasma extravasation induced by Kp-10 was found to be histamine mediated, as co-administration of mepyramine, a histamine H_1_ receptor antagonist [26] , abolished plasma extravasation. An obvious explanation for this phenomenon is Kp-10 induced mast cell degranulation. This is a characteristic shared by substance P in some tissues, whereby a hydrophobic amino acid sequence and terminal basic residue are required to stimulate mast cells, leading to release of histamine and other inflammatory mediators [30]. Accordingly, Kp-10 possesses characteristics similar to these structural elements. Substance P can also cause plasma extravasation via a histamine-independent mechanism, as observed here, providing a positive control in this study [31]. The use of a Kiss1R peptide antagonist p234, was attempted. However this possessed an increased hydrophobocity due to the addition of an N-terminal acetyl group [27], and was also shown to cause plasma extravasation. The lack of pallor ring at the injection site suggests that p234 is unable to stimulate vasoconstriction, most likely due to Kiss1R antagonism. This potentially identifies it as a functioning antagonist, but was not suitable for use locally in skin. Non peptide-based Kiss1R antagonists have been developed which may be more beneficial for future studies [32].

Kp-10 was shown to decrease peripheral blood flow as assessed by ^99m^Tc clearance from injection sites. However, it is noted that in the murine cutaneous microvasculature endothelin-1 is at least 100 times more potent. Similarly, Kp-10 induced plasma extravasation presented with a pallor ring of inhibition at its centre. This is consistent with previous findings whereby Kiss1R activation leads to increases in intracellular Ca^2+^ concentration [33] necessary for contraction of vascular smooth muscle cells [34]. However, inhibition of prostaglandin and thromboxane production by treatment with the cyclooxygenase antagonist indomethacin partially reduced Kp-10 mediated vasoconstriction. Kisspeptins have previously been shown to cause arachidonic acid liberation, the substrate for cyclooxygenases, in CHO cells [33]. The most likely cyclooxygenase-derived mediator of Kp-10-induced decreases in peripheral blood flow is the potent vasoconstrictor thromboxane A_2_ [35]. The vasoconstriction reported in this study supports previous work suggesting kisspeptins cause constriction of several, but not all, human blood vessels [11], but we were unable to assess the contribution of Kiss1R activation to vasoconstriction due to the unsuitability of p234 as an antagonist.

An assessment of *Kiss1* and *Kiss1R* mRNA expression levels in the heart, aorta and kidney was also performed. In agreement with previous studies in humans tissues Kp-10 expression levels were negligible [12], [36], even after treatment with a well known hypertensive agent (angiotensin II) [37]. In contrast, Kiss1R expression was present in all three tissues, but did not increase during hypertension either. This provides evidence that up-regulation of vascular-derived Kp-10 does not play a primary role in the onset of hypertension, although receptors are present in vascular tissues to respond to circulating or locally produced kisspeptin. In support of this concept, a recent publication was not able to provide a positive link between circulating Kp-10 levels and blood pressure changes [38].

Kiss1R has previously been shown to be expressed in the developing embryonic kidney and at birth in mice [39]. However, studies in human tissues have suggested that expression of both *Kiss1* and *Kiss1R* mRNA is high in the brain and placenta, but low in both heart and kidney tissue [33], [36]. Pre-eclampsia, of which both increases and decreases in Kp-10 levels have been observed [14], [15], is a major condition which threatens the health of both mother and child during pregnancy. Expression of Kp-10 protein in syncytiotrophoblasts [4], found at the exchange site of maternal and foetal blood supplies, could influence maternal vascular tone as observed in this study. Additionally, this present data provides evidence for Kp-10 mediated stimulation of mast cell degranulation. Mast cell degranulation has been shown to lead to potent vasoconstriction in isolated placental vascular beds [40]. Furthermore, decreases in placental blood flow has been associated with increases in pre-eclampsia, decreased foetal development and other disorders. Whilst mast cell sub-types are likely to differ between skin and placenta, both are known to be equally stimulated by substance P [41]. Therefore, it is possible that kisspeptins may also be able to activate these mast cells if present in sufficient amounts locally in the placenta. Alternatively, increases in local perivascular production of kisspeptins could contribute to hypertension and plasma extravasation during pregnancy, but also other disorders in which expression of kisspeptins and Kiss1R increase. It is difficult to compare the doses used here with the plasma concentrations observed during pregnancy, but it is possible that the chronic exposure of mothers and foetus to Kp-10 during pregnancy as opposed to the acute, local treatment performed here may elicit the same vascular effects demonstrated.

In conclusion, this is the first study to describe the peripheral microvascular vasoactive properties of kisspeptins. The doses required indicate that these effects may not be of physiological relevance. On the other hand, these effects may become important in conditions where local tissue levels are high, such as in the placenta under certain conditions. Furthermore, knowledge of cardiovascular “off-target” effects could inform the design and application of kisspeptin-based therapeutics, including both antagonists and analogs with improved potency [42].
